# Characterization of a five-microRNA signature as a prognostic biomarker for esophageal squamous cell carcinoma

**DOI:** 10.1038/s41598-019-56367-1

**Published:** 2019-12-27

**Authors:** Jun Yu, Ming Zhu, Min Lv, Xiaoliu Wu, Xiaomei Zhang, Yuanying Zhang, Jintian Li, Qin Zhang

**Affiliations:** 1 Department of Molecular Biology, Jiangsu Cancer Hospital & Jiangsu Institute of Cancer Research & The Affiliated Cancer Hospital of Nanjing Medical University, NO. 42 Baizi Ting, Xuanwu Distinct, Nanjing, 210009 China; 2 Department of Thoracic Surgery, Jiangsu Cancer Hospital & Jiangsu Institute of Cancer Research & The Affiliated Cancer Hospital of Nanjing Medical University, NO. 42 Baizi Ting, Xuanwu Distinct, Nanjing, 210009 China

**Keywords:** Oncogenes, Diagnostic markers

## Abstract

This study aims to identify a miRNAs signature for predicting overall survival (OS) in esophageal squamous cell carcinoma (ESCC) patients. MiRNA expression profiles and corresponding clinical information of 119 ESCC patients were obtained from NCBI GEO and used as the training set. Differentially expressed miRNAs (DEmiRNAs) were screened between early-stage and late-stage samples. Cox regression analysis, recursive feature elimination (RFE)-support vector machine (SVM) algorithm, and LASSO Cox regression model were used to identify prognostic miRNAs and consequently build a prognostic scoring model. Moreover, promising target genes of these prognostic miRNAs were predicted followed by construction of miRNA-target gene networks. Functional relevance of predicted target genes of these prognostic miRNAs in ESCC was analyzed by performing function enrichment analyses. There were 46 DEmiRNAs between early-stage and late-stage samples in the training set. A risk score model based on five miRNAs was built. The five-miRNA risk score could classify the training set into a high-risk group and a low-risk group with significantly different OS time. Risk stratification ability of the five-miRNA risk score was successfully validated on an independent set from the Cancer Genome Atlas (TCGA). Various biological processes and pathways were identified to be related to these miRNAs, such as Wnt signaling pathway, inflammatory mediator regulation of TRP channels pathway, and estrogen signaling pathway. The present study suggests a pathological stage-related five-miRNA signature that may have clinical implications in predicting prognosis of ESCC patients.

## Introduction

According to world statistics, esophageal cancer is among the ten most frequent cancers globally^1^. Its two main histological types are esophageal squamous cell carcinoma (ESCC) and esophageal adenocarcinoma. ESCC is the principal type and accounts for over 90% of esophageal cancer cases in China^2^. Approximately 398,000 ESCCs are reported globally in 2012^3^. It has a poor prognosis, with overall five-year survival of less than 20%^4^.

MicroRNAs (miRNAs) are endogenous small noncoding RNAs, fine-tuning expression of genes in sequence-depending manner^5^. Growing studies have demonstrated that miRNAs play a role in initiation and progression of ESCC through regulating expression of oncogenes and tumor suppressors^6,7^. Prognostic application of miRNAs in ESCC has attracted recent interest. For instance, Chen *et al*. find a prognostic four-miRNA signature through analyzing miRNA expression profile of 119 ESCC samples by microarray^8^. There is evidence that miR-148 is associated with disease-free survival and overall survival (OS) in ESCC patients and could serve as a prognostic biomarker^9^. Additionally, circulating plasma miR-16 and miR-21 are of prognostic value for ESCC patients^10^. Furthermore, a recent study by Mao *et al*. demonstrates potential application of a six-miRNA signature for predicting survival of ESCC patients^11^. However, associations of miRNAs with prognosis of ESCC patients have not been fully elucidated.

Since pathological stage is an important prognostic indicator for ESCC, this study derived a prognostic five-miRNA signature from the differentially expressed miRNAs (DEmiRNAs) between early-stage and late-stage ESCC samples by using Cox regression analysis, recursive feature elimination (RFE)-support vector machine (SVM) algorithm, and LASSO Cox regression model. Moreover, potential target genes of the five miRNAs were predicted and miRNA-target gene networks were built. Function analysis was performed for these target genes to provide insights into the roles played by the five miRNAs in the molecular mechanisms of ESCC.

## Methods

### Retrieval of public data

This study included a training set and a validation set. GSE43732 was used as the training set of this study, including miRNA expression profiles of tumor tissue samples from 119 ESCC patients with clinical information downloaded from National Center for Biotechnology Information (NCBI) Gene Expression Omnibus (GEO) based on Agilent-038166cbc_human_ miR18.0 platform (https://www.ncbi.nlm.nih.gov/geo/queryacc.cgi?Acc=GPL1654). The validation set consisted of miRNA expression data of 93 ESCC tissue samples with the corresponding clinical information downloaded from The Cancer Genome Atlas (TCGA) data portal (https://gdc-portal.nci.nih.gov/; IlluminaHiseq platform).

**Figure 1:**
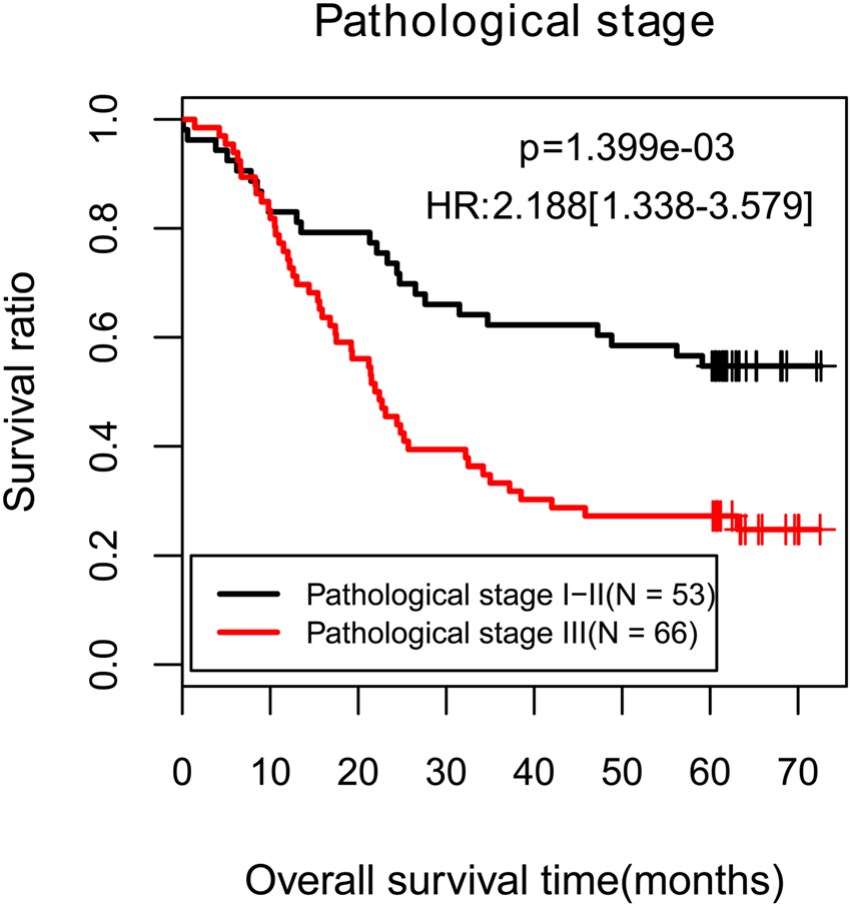
Kaplan-Meier curves for overall survival of patients in GSE43732 classified by pathological stage.

Clinical characteristics of all 119 ESCC patients in GSE43732 were shown in Table 1, and underwent uni-and multi-variate Cox regression analysis by using survival package^12^ of R. The clinical factors with log-rank p-value < 0.05 in uni-variate Cox regression analysis were further included in multi-variate Cox regression analysis. As a result, pathological stage was identified to be an independent prognostic factor (log-rank p < 0.05, Fig. 1 and Table 1) in multi-variate Cox regression analysis. According to pathologic stage, all patients of GSE43732 were separated into an early-stage group (stage I-II) and a late-stage group (stage III).

**Table 1:** Clinicopathological characteristics of ESCC patients and identification of prognostic clinical factors.

Clinical characteristics	GSE43732 (N = 119)	Uni-variate cox regression			Multi-univariate cox regression		
		HR	95%CI	P-value	HR	95%CI	P-value
Age (mean ± SD)	59.03 ± 8.93	1.027	0.999–1.057	0.06	—	—	—
Gender (male/female)	98/21	0.827	0.468–1.461	0.51	—	—	—
Pathologic grade (poorly/moderately/well)	32/64/23	0.819	0.575–1.169	0.27	—	—	—
Pathologic stage (I/II/III)	6/47/66	1.900	1.225–2.946	<0.01	1.512	1.175–2.614	0.01
Pathologic N (N1/N2/N3)	8/20/62/29	1.126	0.838–1.514	0.43	—	—	—
Pathologic T (T0/T1/T2/T3)	54/42/13/10	1.443	1.143–1.821	0.02	1.243	0.915–1.688	0.16
Tumor location (lower/middle/upper)	36/69/14	1.104	0.755–1.614	0.61	—	—	—
Alcohol (yes/no)	74/45	1.053	0.656–1.689	0.83	—	—	—
Tobacco (yes/no/reformed)	80/39	0.859	0.532–1.388	0.54	—	—	—
Death (dead/alive)	46/73	—	—	—	—	—	—
Overall survival days (months, mean ± SD)	37.06 ± 24.25	—	—	—	—	—	—

SD, standard deviation.

### Screening DEmiRNAs between early-stage and late-stage samples

MiRNA-expression data of GSE43732 and the TCGA set was subject to unit scale normalization and median scale normalization as described previously^13^. Briefly, unit scale normalization was aimed to acquire samples with a norm scaled to 1 using the following formula:

For a given sample vector *v* = (*v*_1_, …, *v*_*n*_)

$${v}_{normed}=v\,.\frac{1}{{v}_{2}^{2}}$$

${\Vert v\Vert }_{2}^{2}$ means the *l*_2_ norm of *v*, which is obtained using sqrt(sum(data^2^)) function of R.

Median scale normalization was conducted using the following two equations.

For a give feature vector *x* = (*x*_1_, …, *x*_*n*_):

$$mad(x)=median(\{|{x}_{i}-median(x)|,{x}_{i}\in x\})$$

The median absolute deviation (*mad*) is used to estimate variability of a uni-variate sample.

$${x}_{scaled}=(x-median(x)).\frac{1}{mad(x)}$$

Following data normalization, differential expression analysis was performed between early-and late-stage samples in GSE43732 using limma package https://bioconductor.org/packages/release/bioc/html/limma.html) of R. The miRNAs with FDR < 0.05 and |log_2_FC| > 0.263 were identified as significant DEmiRNAs, followed by two-way hierarchical clustering analysis based on centered pearson correlation^14^ algorithm with pheatmap package of R.

### SVM analysis

We employed survival package of R to conduct uni-variate Cox regression analysis to select the miRNAs significantly associated with OS in GSE43732 from the pre-selected DEmiRNAs, using log-rank p < 0.05 as the cutoff. Subsequently, we identified optimal feature miRNAs from these OS-related miRNAs within GSE43732 set by implementing RFE algorithm with caret package^15^ of R. Finally, SVM classifier (core: Sigmoid Kernel; cross: 100-fold cross validation) was built using the optimal feature miRNAs with SVM function^16^ of e1071 package of R. The classifier was used to distinguish early-stage samples from late-stage samples in GSE43732 and the TCGA set, respectively. Efficacy of the classifier was evaluated by concordance index (C-index)^17^, Brier score^18^, logRank p-value of cox-PH regression, and a number of receiver operating characteristic (ROC) curve-related metrics including area under ROC curve (AUROC), sensitivity, specificity, positive predictive value (PPV) and negative predictive value (NPV).

### Definition of a prognostic scoring model

In order to build a risk scoring model for survival prediction in ESCC patients, initially, we used the fore-mentioned optimal feature miRNAs to fit the LASSO Cox regression model for identification of optimal predictive miRNAs by employing penalized package of R. Optimal lambda value was calculated via a 1,000 cross-validations. Consequently, LASSO Cox regression coefficients and expression levels of the identified optimal predictive miRNAs were used to establish a prognostic scoring model as follows:

$${\rm{Risk}}\,{\rm{score}}=\sum \,{{\rm{coef}}}_{{\rm{DEmiRNAs}}}\times {\mathrm{Exp}}_{{\rm{DEmiRNAs}}}$$

Using the prognostic model, risk scores were calculated for all patients within GSE43732. Using median risk score as the threshold, GSE43732 was separated into a high-risk group and a low-risk group. The two risk groups were compared for OS by using Kaplan-Meier curves^19^ and log-rank test. ROC curves were applied to estimate predictive value of the miRNAs-based prognostic model. To validate prognostic capability of the model in the TCGA set, similarly, the TCGA set was divided into a high-risk group and a low-risk group according to median risk score. Similarly, OS of the two risk groups was compared using Kaplan-Meier and log-rank methods.

### Function analysis

As described above, the TCGA set was dichotomized by risk score into a high-risk group and a low-risk group. Using paired mRNA-seq data of miRNA-seq data of all 93 ESCC samples in the TCGA set, we screened differentially expressed genes (DEGs) between the two risk groups of the TCGA set. The cutoff for selection of DEGs was set at FDR < 0.05 and |log_2_FC| > 0.263. Besides, target genes of the identified prognostic miRNAs were predicted using starBase V3.0^20^ (a http://starbase.sysu.edu.cn/). We reserved the target genes that were identified by at least one of five miRNA target prediction programs^21^ including targetScan (http://www.targetscan.org), picTar (http://pictar.mdc-berlin.de/), RNA22 (http://cbcsrv.watson.ibm.com/rna22.html), PITA (http://genie.weizmann.ac.il/pubs/mir07/) and miRanda (http://www.microrna.org/microrna/home.do). The overlapped genes between the identified target genes and the selected DEGs were chosen to construct a miRNA-target gene network with these prognostic miRNAs using Cytoscape software^22^. For genes in the network, gene ontology (GO)^23^ function and Kyoto Encyclopedia of Genes and Genomes^24^ pathway enrichment analyses were performed using database for annotation, visualization, and integrated discovery (DAVID)^25^ tool. When p-value <0.05, a GO term or a pathway was considered significant.

## Results

### Identification of DEmiRNAs

**Figure 2:**
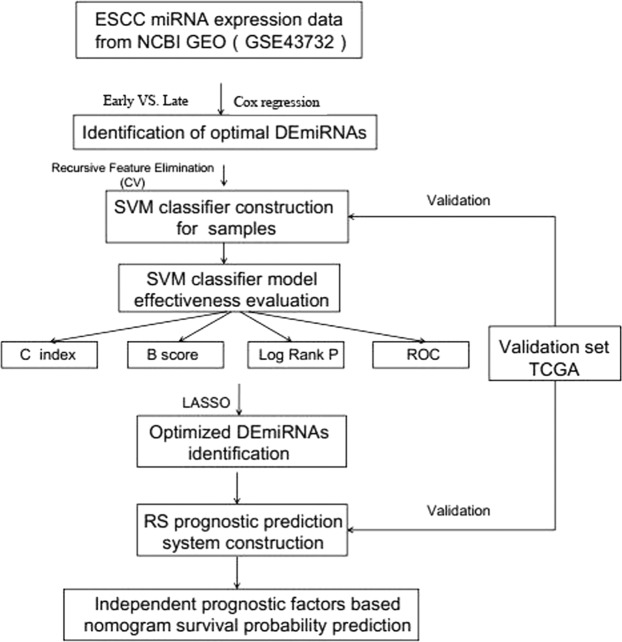
Graphic demonstration of DEmiRNAs. (**A**) Volcano plot of effect size (log_2_FC) and −log_10_(FDR) of miRNAs. Blue round spots represent DEmiRNAs, and black round spots represent non-DEmiRNAs. Horizontal dash line indicates FDR < 0.05, and two vertical dash lines indicate |logFC| > 0.263. (**B**) A heatmap for two-way hierarchical clustering analysis of DEmiRNAs.

**Figure 3:**
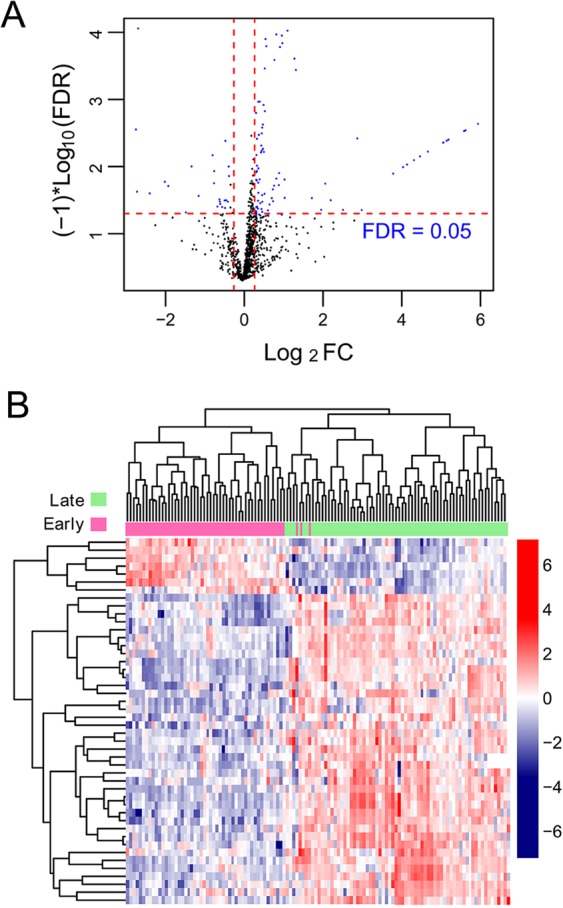
RFE algorithm optimization process. The horizontal axis is the number of selected miRNAs, and the vertical axis is cross-validation accuracy. When 10 feature miRNAs are selected, the model has the highest accuracy.

Overall study design was depicted in Fig. 2. GSE43732 (training set) comprised of 53 early-stage (stage I-II) samples and 66 late-stage (stage III) samples. MiRNAs expression profiles of these samples were analyzed. A total of 46 DEmiRNAs between the early-stage and late-stage samples were acquired, consisting of 6 down-regulated miRNAs and 40 up-regulated miRNAs in the late-stage samples compared to the early-stage samples (Fig. 3A). The two-way hierarchical- clustering heatmap showed that expression patterns of these DEmiRNAs were obviously different in the early-stage and late-stage samples (Fig. 3B).

### Definition of a SVM classifier of 10 miRNAs

**Figure 4:**
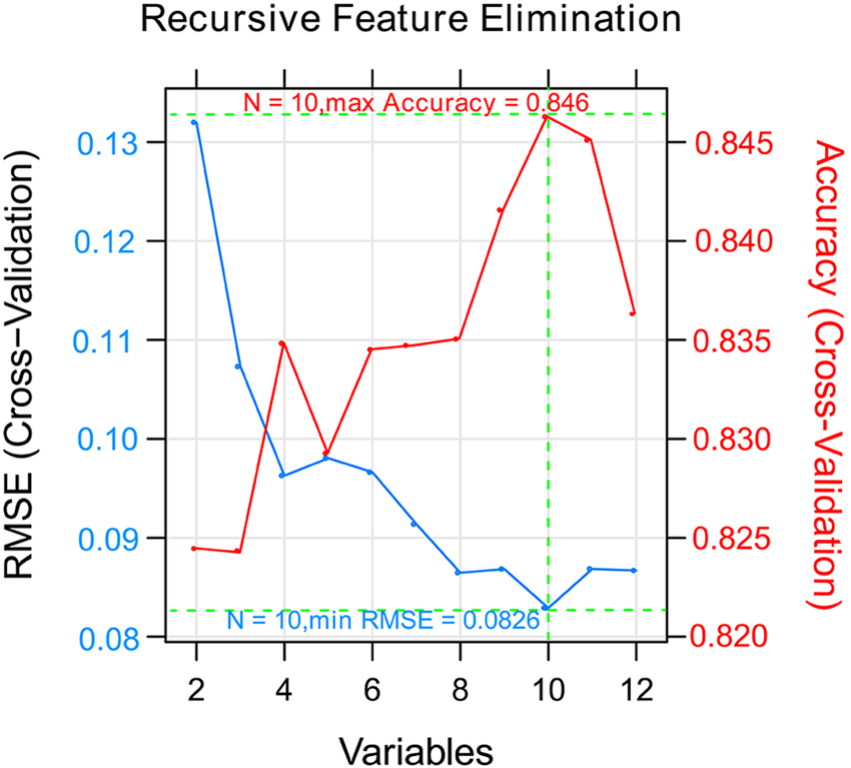
Scatter plot for early-stage and late-stage samples predicted by the SVM classifier in the training set (**A**) and the validation set (**B**).

Using data of GSE43732, we performed uni-variate Cox regression analysis to identify OS-related miRNAs from the identified 46 DEmiRNAs. As a result, 12 significant miRNAs (log-rank p < 0.05) were obtained. We then applied the RFE algorithm to filter the 12 OS-related miRNAs in order to identify the optimal combination of feature miRNAs in GSE43732. Finally, 10 miRNAs (maximal accuracy = 0.846, minimal RMSE = 0.0826) were identified as the optimal feature miRNAs to construct the classification model using an SVM (Fig. 4).

**Figure 5:**
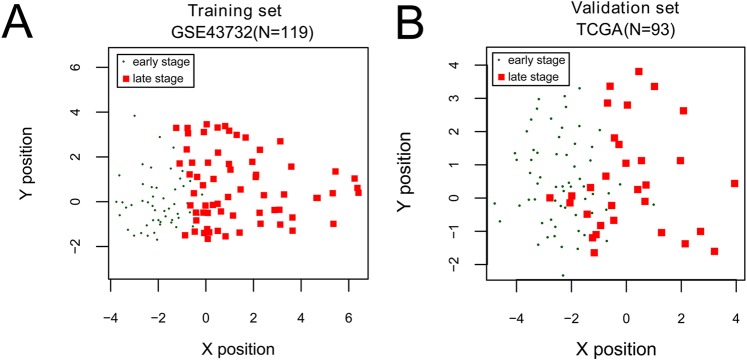
Kaplan-Meier survival curves (left) and ROC curves (right) to evaluate the SVM classification model based on ten miRNAs in the training set (**A**) and the validation set. (**B**) Patients are stratified into an early-stage group and a late-stage group by the classification model.

**Figure 6:**
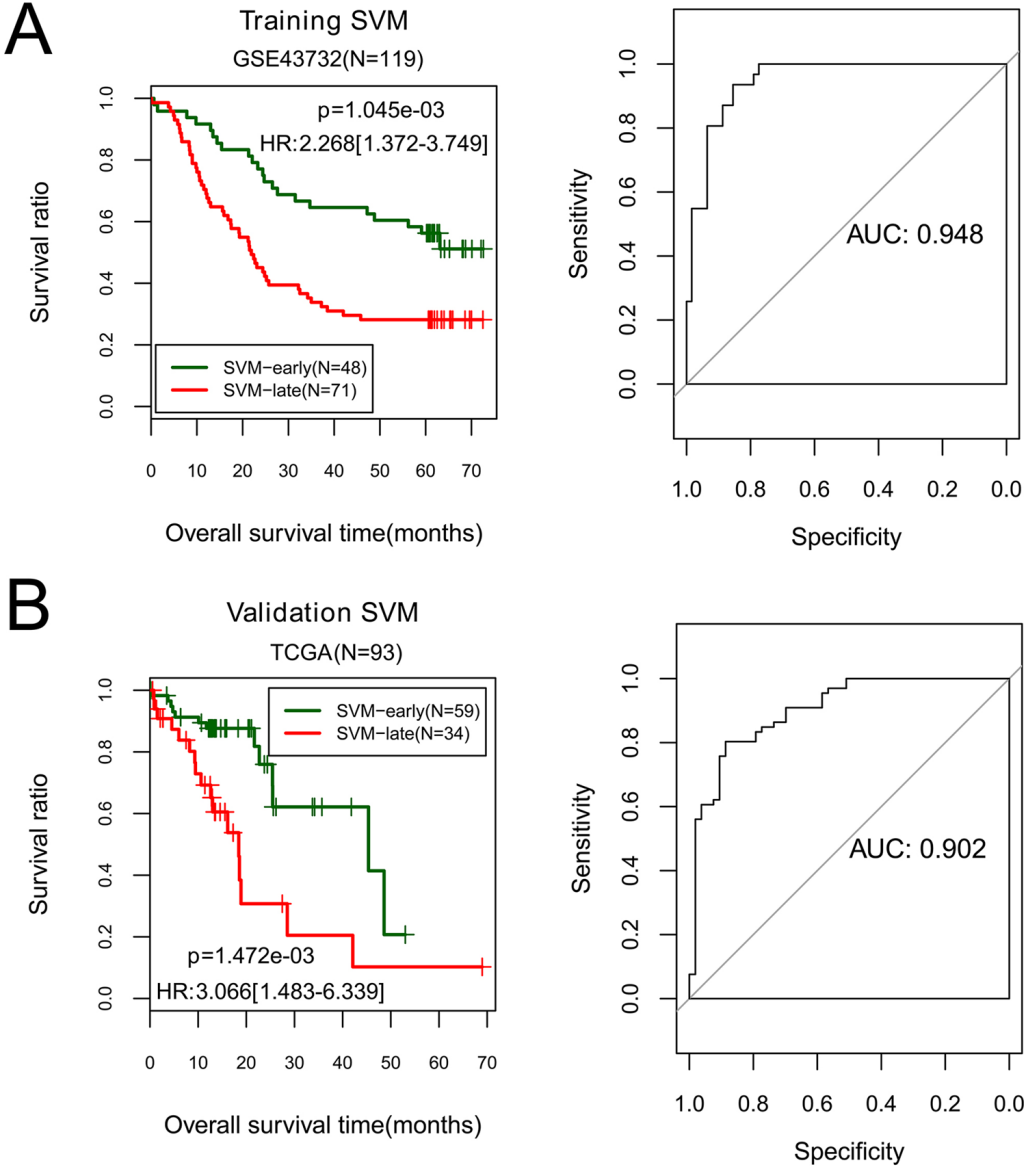
Kaplan-Meier survival curves (left) and ROC curves (right) to evaluate the five-miRNA prognostic signature in the training set (**A**) and the validation set. (**B**) Patients are separated into a high-risk group and a low-risk group by risk score.

The constructed SVM classifier was applied on both GSE43732 and the TCGA set (validation set). As shown in Fig. 5A,B, the classifier could successfully differentiate early-stage samples from late-stage samples in both two datasets. Moreover, there was significant difference in OS time between predicted early-stage and late-stage samples in both two datasets (GSE43732, p-value = 1.05E^−03^; TCGA set, p-value = 1.47E^−03^, Fig. 6A,B). GSE43732 generated C-index of 0.867, Brier score of 0.0542 and AUROC of 0.948, while the TCGA set generated C-index of 0.819, Brier score of 0.0962 and AUROC of 0.902 (Table 2, Fig. 6A,B). These results illustrate that the classification model based on the 10 miRNAs could accurately discriminate between early-stage and late-stage ESCC samples.

**Table 2:** Effectiveness evaluation of the classifier of ten miRNAs on training set and validation set.

Datasets	C index	Brier score	Log rank P	ROC				
				AUROC	Sensitivity	Specificity	PPV	NPV
Training set (GSE43732, N = 119)	0.867	0.054	1.05 × 10^−3^	0.948	0.755	0.879	0.833	0.817
Validation set (TCGA, N = 93)	0.819	0.096	1.47 × 10^−3^	0.902	0.806	0.710	0.847	0.747

ROC, receiver operating characteristic curve; AUROC, area under the receiver operating characteristic curve; PPV, positive predictive value; NPV, negative predictive value.

### Construction of a prognostic scoring model based on five miRNAs

To construct a risk score model for predicting survival in ESCC, we used the above-mentioned 10 feature miRNAs to fit the LASSO Cox regression model. With parameter lambda of 8.153 obtained by performing 1000 cross-validations, we identified a prognostic panel of five miRNAs (Table 3), including miR-181c-5p, miR-195-5p, miR-203, miR-212-3p and miR-28-5p. A prognostic prediction model was developed based on the five miRNAs as follows:

$$\begin{array}{rcl}{\rm{Risk}}\,{\rm{score}} & = & (0.0368)\times {\mathrm{Exp}}_{{\rm{hsa}} \mbox{-} {\rm{mir}}181{\rm{c}} \mbox{-} 5{\rm{p}}}\\  &  & +(0.0831)\times {\mathrm{Exp}}_{{\rm{hsa}} \mbox{-} {\rm{mir}} \mbox{-} 195 \mbox{-} 5{\rm{p}}}+(\,-\,0.1629)\\  &  & \times {\mathrm{Exp}}_{{\rm{hsa}} \mbox{-} {\rm{mir}} \mbox{-} 203}+(0.0894)\times {\mathrm{Exp}}_{{\rm{hsa}} \mbox{-} {\rm{mir}} \mbox{-} 212 \mbox{-} 3{\rm{p}}}\\  &  & +(0.0489)\times {\mathrm{Exp}}_{{\rm{hsa}} \mbox{-} {\rm{mir}} \mbox{-} 28 \mbox{-} 5{\rm{p}}}\end{array}$$

**Table 3:** The five-miRNA signature for survival prediction.

miRNA	coefficient	Hazard ratio	95%CI	P-value
miR-181c-5p	0.0368	1.129	1.085–1.510	0.041
miR-195-5p	0.0831	1.116	1.083–1.506	0.0471
miR-203	−0.1629	0.789	0.618–0.906	0.0156
miR-212-3p	0.0894	1.204	1.135–1.551	0.0151
miR-28-5p	0.0489	1.095	1.012–1.493	0.0454

**Figure 7:**
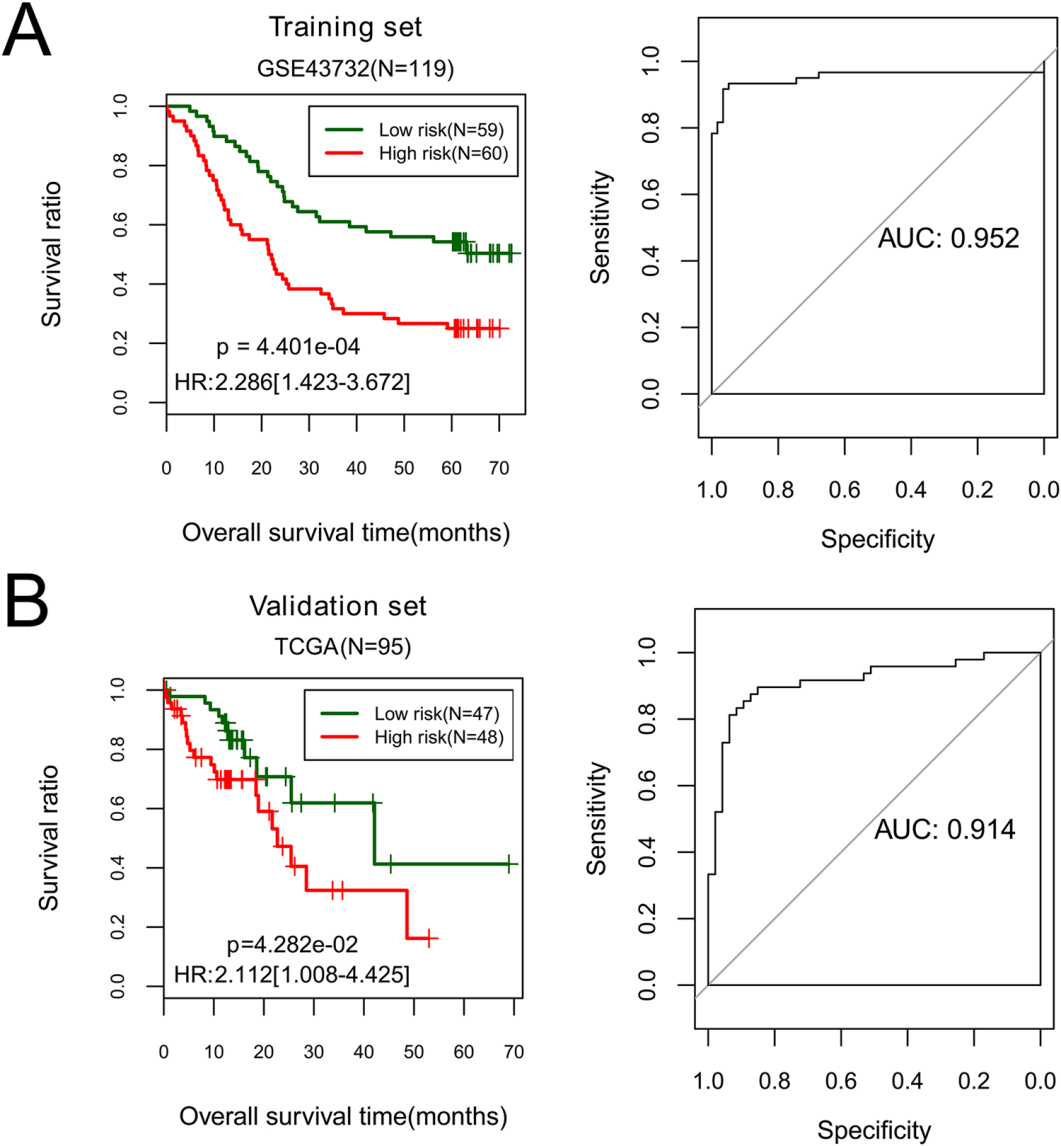
A nomogram combining pathological stage and RS status for predicting prognosis of ESCC patients. (**A**) total points of pathological stage and RS status are used to decide probability of 5-year OS of each individual patient. (**B**) Calibration plots of nomogram for predicting 5-year OS.

With median risk score as threshold, GSE43732 was divided into a low-risk group and a high-risk group. OS time was significantly different between the two risk groups (p-value = 4.401E^−04^), with AUC of 0.952. These results suggest that the five-miRNA risk score could predict survival in ESCC patients. Moreover, the TCGA set was separated by the risk score model into two risk groups with significantly different OS time (p-value = 4.282E^−02^, AUC = 0.914, Fig. 7). These observations showed that prognostic value of the five-miRNA risk score was successfully validated in the TCGA set.

### Functional annotation for target genes of the five prognostic miRNAs

**Figure 8:**
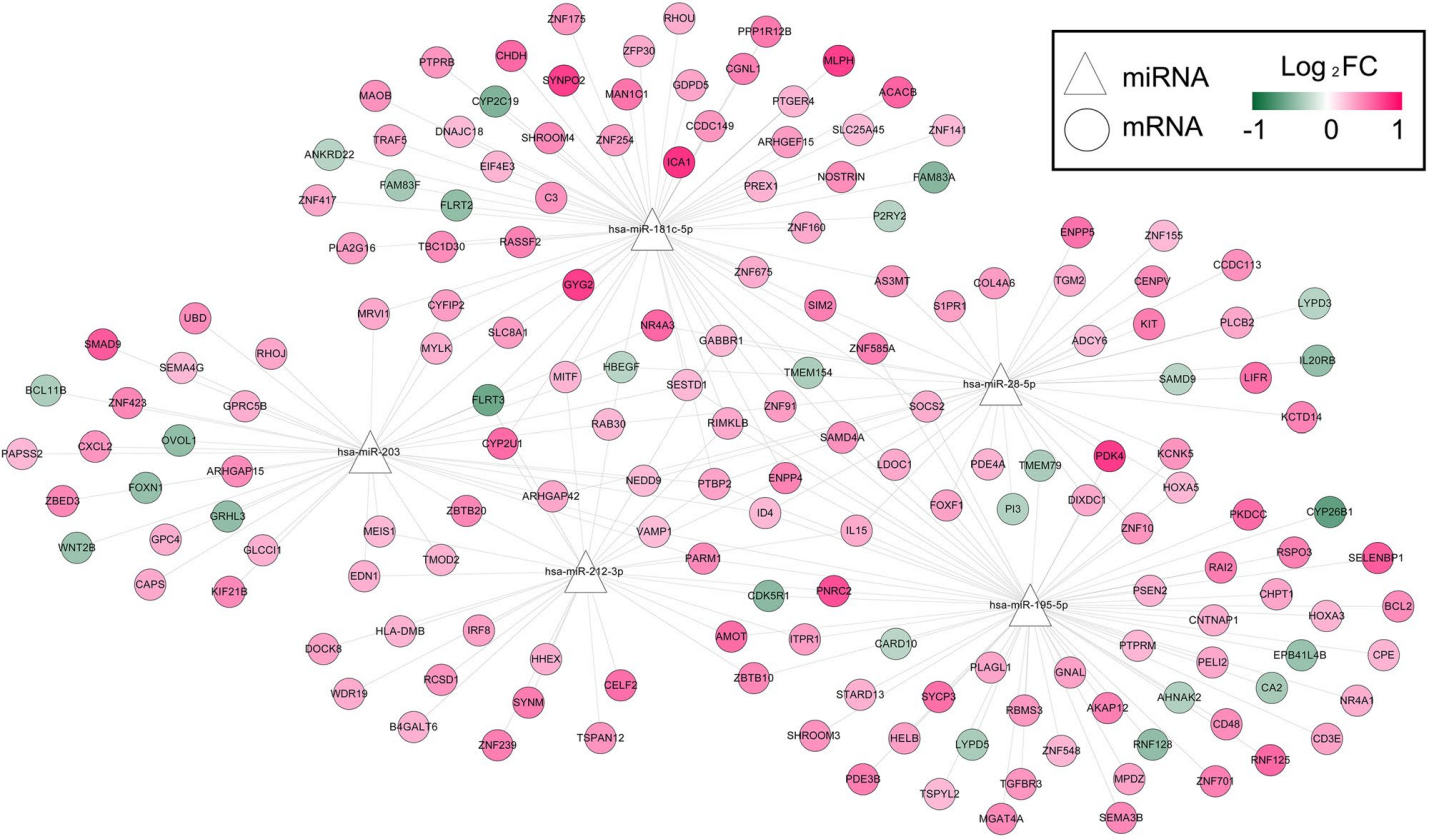
MiRNA-mRNA networks. Triangular nodes stand for miRNAs, and round nodes stand for mRNAs. Colors of nodes from green to red indicate values of log_2_FC.

Using paired mRNA-seq data of miRNA-seq data of the TCGA set, 684 DEGs were found between the high-risk and low-risk samples of the TCGA set (FDR < 0.05 and log_2_FC| > 0.263), consisting of 526 up-regulated genes and 158 down-regulated genes in the high-risk samples relative to the low-risk samples. Out of these DEGs, the genes that were predicted to be target genes of the five prognostic miRNAs by using StarBase software were reserved. A total of 228 miRNA-target gene pairs were obtained and then used to construct a miRNA-target gene network (Fig. 8). The genes in the network were functionally related to 18 GO biological process (BP) terms, such as positive regulation of inflammatory response and Wnt signaling pathway (Table 4). Regarding KEGG pathways, cGMP-PKG signaling pathway, gastric acid secretion, vascular smooth muscle contraction, inflammatory mediator regulation of TRP channels, estrogen signaling pathway, pathways in cancer and aldosterone synthesis and secretion were important for these genes (Table 4).

**Table 4:** Results of GO function and KEGG pathway enrichment analyses.

Category	Term	Count of genes	P-value
GO biology process	Cytokine-mediated signaling pathway	8	2.70 × 10^−4^
	Positive regulation of cell migration	8	2.00 × 10^−3^
	Negative regulation of angiogenesis	5	2.95 × 10^−3^
	Negative chemotaxis	4	4.16 × 10^−3^
	Establishment of skin barrier	3	1.26 × 10^−2^
	Melanocyte differentiation	3	1.55 × 10^−2^
	Signal transduction	20	1.56 × 10^−2^
	Thyroid gland development	3	2.37 × 10^−2^
	Mesenchymal-epithelial cell signaling	2	2.85 × 10^−2^
	Cornification	2	2.85 × 10^−2^
	Transcription, DNA-templated	28	3.24 × 10^−2^
	Positive regulation of inflammatory response	4	3.30 × 10^−2^
	Wnt signaling pathway	6	3.43 × 10^−2^
	Blood vessel remodeling	3	3.76 × 10^−2^
	Lung alveolus development	3	4.20 × 10^−2^
	Regulation of cell shape	5	4.59 × 10^−2^
	Positive regulation of glucose transport	2	4.70 × 10^−2^
	Negative regulation of cell cycle	3	4.89 × 10^−2^
KEGG pathway	cGMP-PKG signaling pathway	7	5.21 × 10^−3^
	Gastric acid secretion	5	6.31 × 10^−3^
	Vascular smooth muscle contraction	6	6.56 × 10^−3^
	Inflammatory mediator regulation of TRP channels	5	1.73 × 10^−2^
	Estrogen signaling pathway	5	1.79 × 10^−2^
	Pathways in cancer	9	4.50 × 10^−2^
	Aldosterone synthesis and secretion	4	4.90 × 10^−2^

GO, gene ontology; KEGG, Kyoto Encyclopedia of Genes and Genomes.

## Discussion

EC is among the five most common causes of cancer-related death in China^26^. ESCC is the most common subtype of EC in China^27^. Accumulating evidence reveals that investigating esophageal carcinogenesis-related miRNAs is potentially useful for developing prognostic biomarkers^28,29^. The present study used miRNA expression profiles of ESCC samples from GEO to identify prognostic miRNAs. Total 46 DEmiRNAs were found between the early-stage and late-stage samples. A classification model based on 10 miRNAs for pathological stage was built by using RFE-SVM method. The SVM classifier performed well in classifying all ESCC samples into early-stage and late-stage groups on both GSE43732 set and the TCGA set, as evidenced by values of C-index, Brier score, AUROC, sensitivity, specificity, PPV and NPV. Moreover, by using LASSO Cox regression model, five prognostic miRNAs (miR-181c-5p, miR-195-5p, miR-203, miR-212-3p and miR-28-5p) were identified. The five-miRNA risk score could dichotomize the training set (GSE43732 set) into two risk groups with significantly different OS time. Moreover, prognostic capability of the five-miRNA risk score was successfully confirmed in an independent validation set (TCGA set).

Down-regulated miR-181c-5p is found in EC tissues compared to adjacent normal tissues^30^. MiR-181c-5p is observed to be dys-regulated in patients with pancreatic cancer relative to healthy controls and be significantly up-regulated in pancreatic cancer cases in comparison with chronic pancreatitis controls^31^. Up-regulation of miR-195-5p is reported in plasma of patients with laryngeal squamous cell carcinoma compared to healthy subjects^32^. Circulating miR-195-5p has been found to serve as a promising prognostic biomarker in head and neck cancer patients, with high expression indicative of poor prognosis^33^. There is evidence that miR-203 is associated with OS in esophageal adenocarcinoma patients^9^. Thomas *et al*. report that miR-203 has an oncogenic activity in pancreatic cancer and maybe a prognostic biomarker^34^. Down-regulated expression of miR-212-3p is related to radio-resistance in nasopharyngeal carcinoma^35^. Liu *et al*. suggest that miR-212-3p exerts an inhibitory effect on glioblastoma cell proliferation through targeting serum and glucocorticoid-inducible kinase 3^36^. miR-28-5p is observed to be decreased in serum and tumor specimens of patients with renal cell carcinoma, and plays a tumor-suppressive role in this cancer^37,38^. To the best of our knowledge, this is the first time that the five-miRNA signature is suggested for survival prediction of ESCC patients. The prognostic model based on the five-miRNA signature enables differentiation of patients at high risk of mortality from patients at low risk of mortality, thus paving a way for development of personalized therapies.

To gain an understanding of functional roles of the five signature miRNAs in ESCC, we constructed a miRNA-target gene network using these miRNAs and their target genes, and analyzed possible biological processes and signaling pathways that involve these target genes. Results showed that the five miRNAs might be functionally related to Wnt signaling pathway, cGMP-PKG signaling pathway, inflammatory mediator regulation of TRP channels pathway, several inflammation- related biological processes, and estrogen signaling pathway. Abnormal activation of WNT signaling pathway contributes to esophageal tumorigenesis^39^. Recently, shi *et al*. uncover that up-regulated circRNAs are implicated in cGMP-PKG signaling pathway in ESCC^40^. TRP channels are associated with tumorigenesis and may represent promising therapeutic targets^41^. TRPC6 channel highly expressed in ESCC is critical for cell proliferation and cell cycle^42^. It has been established that inflammation plays an important role in cancer progression^43^. Several inflammatory biomarkers, such as neutrophil/lymphocyte ratio, platelet/lymphocyte ratio and lymphocyte/monocyte ratio have shown prognostic value in ESCC patients^44^. A recent study demonstrates that estrogen suppresses proliferation of human ESCC cells via estrogen-Ca^2+^ signaling pathway^45^. Nevertheless, further investigations are necessary to confirm these findings of our study.

## Conclusion

In summary, our study identified a pathological stage-related five-miRNA signature as a promising predictor of OS for ESCC patients. Several biological processes and signaling pathways are unveiled to show that these miRNAs may participate in various molecular mechanisms of ESCC. Other independent cohorts of large sample size are needed to further validate prognostic value of the five-miRNA signature in ESCC.
